# Utilizing innovative two curves in nomogram

**DOI:** 10.3389/fmed.2024.1478603

**Published:** 2025-01-07

**Authors:** Tianhan Zhou, Zhongkai Ni, Hao Fan, Hai Huang, Haimin Jin

**Affiliations:** ^1^The Department of General Surgery, Hangzhou TCM Hospital Affiliated to Zhejiang Chinese Medical University, Hangzhou, China; ^2^School of Clinical Medicine, Zhejiang Chinese Medicine University, Hangzhou, China

**Keywords:** nomogram, cutoff points, threshold, clinical prediction model, Youden index

## Abstract

**Objective:**

Nomograms are valuable tools in clinical research for predicting patient outcomes. Understanding threshold values within these models is crucial for assessing the model’s effectiveness and practical application in clinical environments.

**Methods:**

We developed two novel interpretive curves to enhance the utility of nomograms. These curves were designed to provide clear visualization of how clinical prediction models perform across various thresholds. The curves are applied to two case studies to demonstrate their practical application and efficacy.

**Results:**

In both examples, the novel curves successfully highlighted critical threshold values and revealed changes in prediction accuracy across these thresholds. This enhanced the understanding of the nomogram’s performance, providing clinicians with more informative decision-making tools.

**Conclusions:**

The introduction of these interpretive curves allows for a more nuanced understanding of nomogram-based predictions, offering insights into threshold effects that can inform clinical decisions.

## Introduction

Clinical prediction models encompass the utilization of parametric, semi-parametric, or non-parametric models to evaluate the probability of experiencing a specific outcome in the future (1). These models are typically classified into diagnostic models, prognostic models. As quantitative instruments for analysis and benefit assessment, clinical prediction models offer more objective and precise information to aid physicians, patients, and healthcare administrators in their decision-making processes (2). The ability to predict clinical outcomes is of seminal importance in the physician-patient relationship. For physicians, the ability to understand the most likely end point of a patient’s clinical course may allow the modification of disease surveillance and treatment in such a way that improved outcomes can be achieved (3).

Nomograms have long been indispensable tools in the realm of clinical prediction modeling, offering a unique blend of simplicity and precision in healthcare decision-making (4). Nomograms are products of rigorous statistical analysis. The construction process involves the derivation of mathematical equations, often through methods like logistic regression or Cox proportional hazards modeling, that capture the impact of each variable on the predicted outcome. These equations are then translated into the graphical elements of the nomogram, ensuring that it remains a clinically relevant and robust decision support tool (5). These graphical instruments provide an intuitive means of estimating the probability of specific clinical outcomes based on the interplay of multiple predictive variables, rendering complex statistical models accessible in real world scenarios.

However, nomograms typically offer probabilities for forecasting outcome variables, necessitating additional input from clinical physicians to make informed decisions. Additionally, the predictive efficacy of clinical prediction models fluctuates significantly across various thresholds (6). The variability of clinical prediction models’ predictive performance across various thresholds is a noteworthy consideration. In order to provide a more comprehensive understanding of the model’s accuracy, sensitivity, and specificity at different thresholds, we constructed cutoff value selection curves and nomogram evaluation curves. These visual aids serve to elucidate the internal characteristics of the clinical prediction model and facilitate more effective resolution of clinical queries (4).

Clinical prediction models encompass the utilization of parametric, semi-parametric, or non-parametric models to evaluate the probability of an individual’s present affliction with a particular disease or the probability of experiencing a specific outcome in the future (1). These models are typically classified into diagnostic models, prognostic models. As quantitative instruments for analysis and benefit assessment, clinical prediction models offer more objective and precise information to aid physicians, patients, and healthcare administrators in their decision-making processes (2). The ability to predict clinical outcomes is of seminal importance in the physician-patient relationship. For physicians, the ability to understand the most likely end point of a patient’s clinical course may allow the modification of disease surveillance and treatment in such a way that improved outcomes can be achieved (6).

Nomograms have long been indispensable tools in the realm of clinical prediction modeling, offering a unique blend of simplicity and precision in healthcare decision-making. Nomograms are products of rigorous statistical analysis. The construction process involves the derivation of mathematical equations, often through methods like logistic regression or Cox proportional hazards modeling, that capture the impact of each variable on the predicted outcome. These equations are then translated into the graphical elements of the nomogram, ensuring that it remains a clinically relevant and robust decision support tool. These graphical instruments provide an intuitive means of estimating the probability of specific clinical outcomes based on the interplay of multiple predictive variables, rendering complex statistical models accessible in real world scenarios.

However, nomograms typically offer probabilities for forecasting outcome variables, necessitating additional input from clinical physicians to make informed decisions. Additionally, the predictive efficacy of clinical prediction models fluctuates significantly across various thresholds (6). The variability of clinical prediction models’ predictive performance across various thresholds is a noteworthy consideration. In order to provide a more comprehensive understanding of the model’s accuracy, sensitivity, and specificity at different thresholds, we constructed cutoff value selection curves and nomogram evaluation curves. These visual aids serve to elucidate the internal characteristics of the clinical prediction model and facilitate more effective resolution of clinical queries (7).

### Cutoff value selection curve

**Figure 1:**
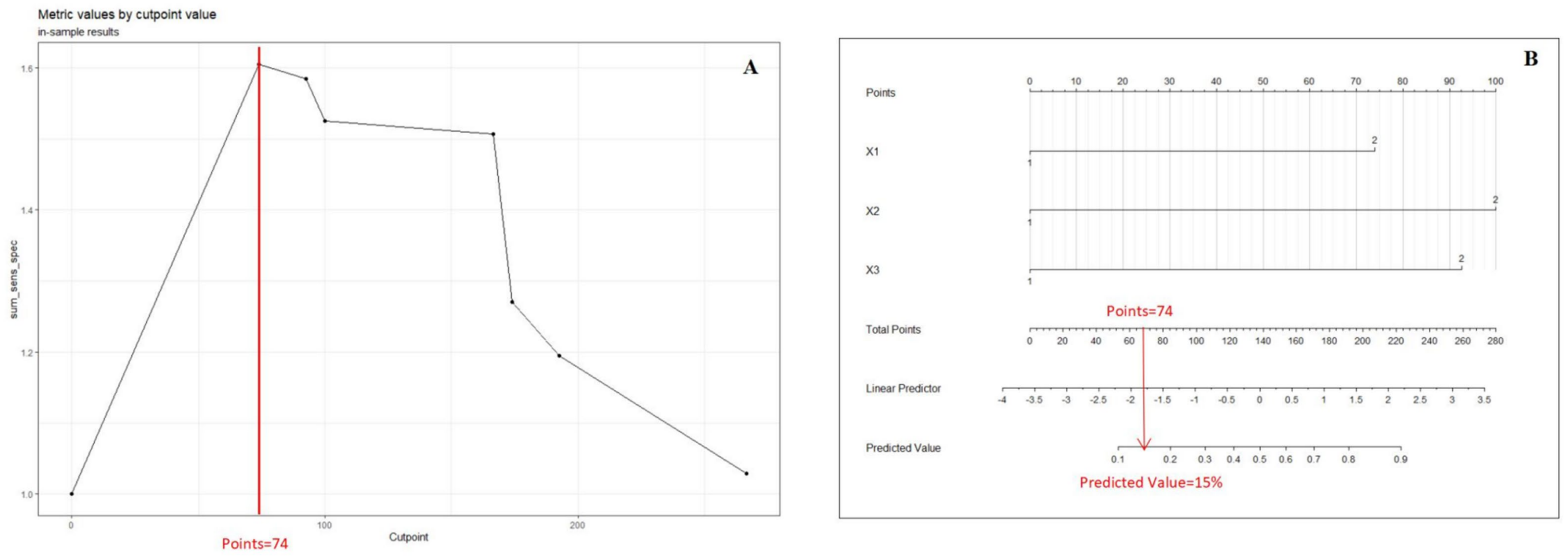
**(A)** Cutoff value selection curve shows the nomogram by illustrating the correlation between total points and the Youden Index. **(B)** Presents the nomogram at the optimal cutoff value. When the Youden Index is maximized, the cutoff value is 74, and the threshold for the prediction score is set at 15%.

The curve to determine the threshold value of the nomogram was constructed using the “cutpointr” package in R, aiming to predict the total score on the nomogram. This package is specifically designed for identifying cutoff points for continuous variables and offers a practical approach to discretizing such variables into ordered categorical variables (8). The “cutpointr” package facilitates automatic determination of cutoff points by utilizing specific statistical metrics, such as maximizing sensitivity, specificity, or the Youden index. These cutoff points help bifurcate continuous variables into ordered categorical variables, improving classification accuracy. In our study, a clinical prediction model was constructed utilizing variables X1, X2, and X3 to forecast the outcome Y. The Cutoff value selection curve was plotted to optimize sensitivity and the total score, resulting in the determination of a cutoff value of 74 and a predicted percentage of 15% (Figure 1). It is noteworthy that the determination of the cutoff value for a nomogram is contingent upon the particular context in which it is employed and the requisite balance between sensitivity and specificity within that context. Consequently, the cutoff value may exhibit variability contingent upon the intended utilization of the nomogram.

### Evaluation curve for predictive efficacy

**Figure 2:**
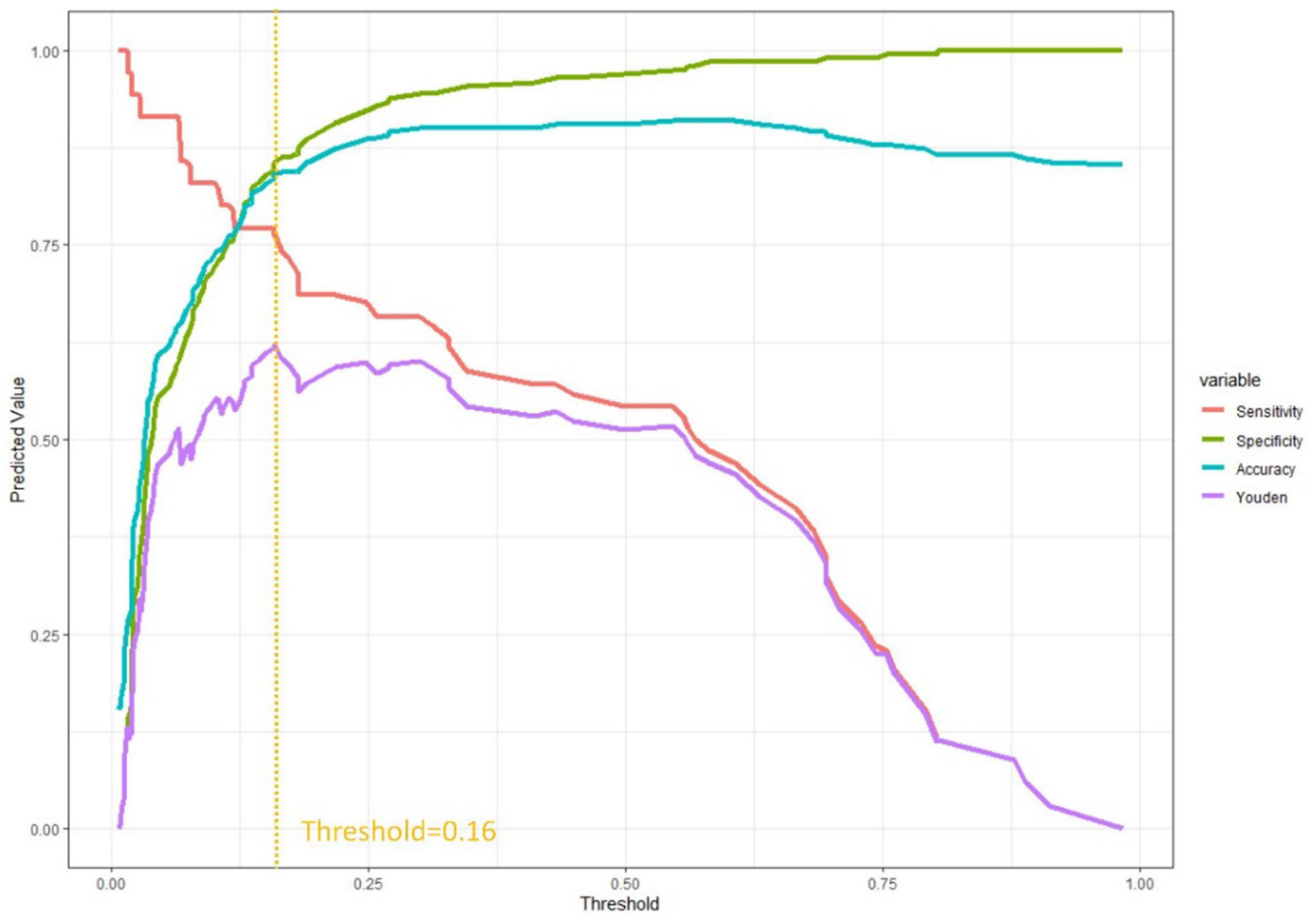
Evaluation curve for predictive efficacy shows when the threshold was at 0.16, the model shows better sensitivity and specificity.

In order to provide additional evidence of the clinical prediction model’s predictive efficacy across various thresholds, we developed an evaluation curve to depict its performance. The curve aptly illustrates the variations in the model’s predictive efficacy at distinct thresholds, thereby offering a lucid depiction of the model’s performance across diverse threshold levels. The present study employed the statistical software R to construct a threshold-based for loop, whereby the Youden index, sensitivity, specificity, and accuracy of the prediction model were computed across varying thresholds. A graphical representation of the threshold values on the x-axis and the corresponding alterations in the aforementioned indices on the y-axis was generated using the ‘ggplot2’ package (Figure 2). Our example attains the highest Youden index at a combined predicted value of 0.16, signifying an optimal equilibrium between sensitivity and specificity. The combined index denotes the value derived from the collective prediction of multiple factors.

### Two curves in nomograms for informed clinical decisions

**Figure 3:**
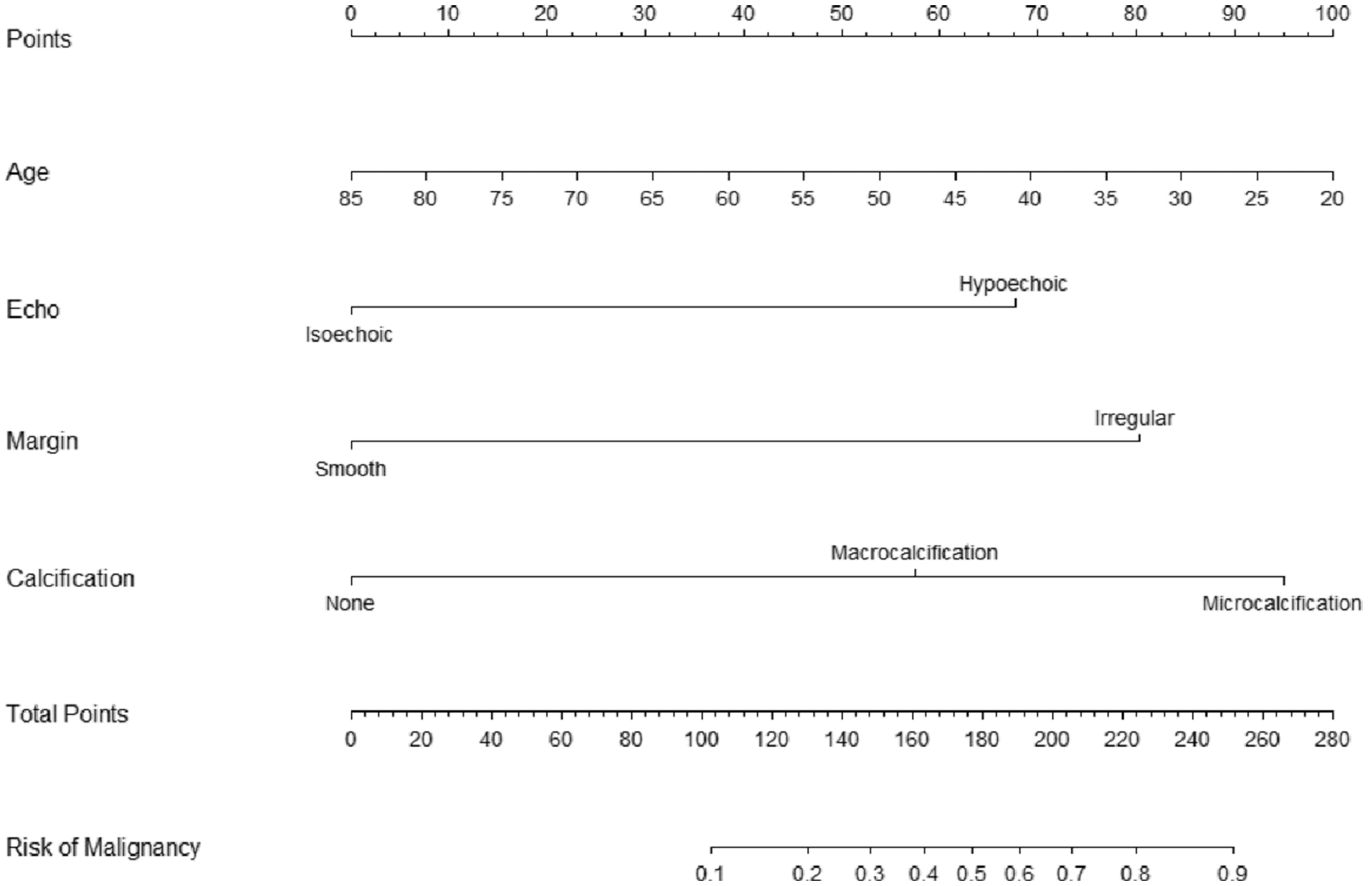
Nomogram for predicting malignancy of PCTNs.

**Figure 4:**
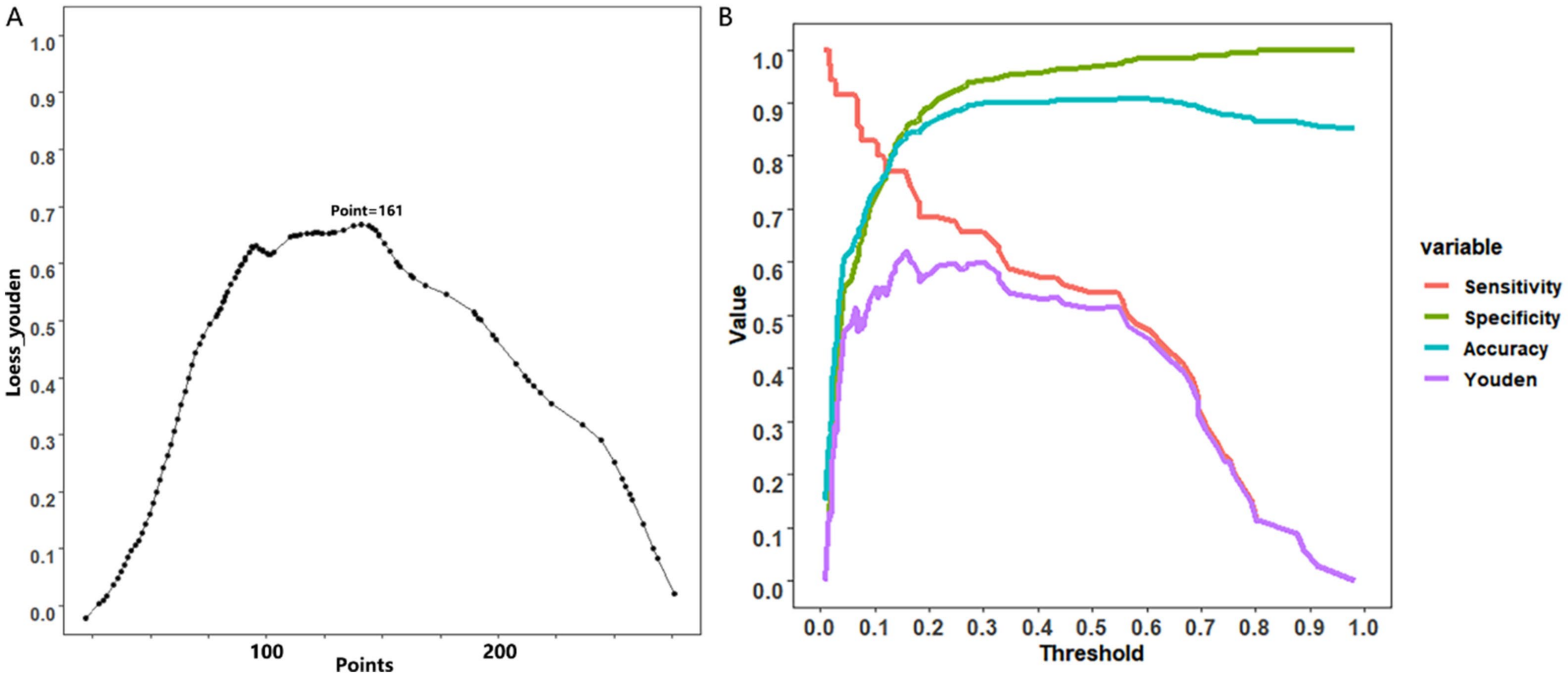
Cutoff value selection curve **(A)** and evaluation curve for predictive efficacy **(B)** were used in nomogram for predicting malignancy of PCTNs.

The utilization of nomograms allows for precise computation of a cumulative score based on individual patient characteristics, providing corresponding probabilities for various outcomes. For instance, consider a patient named A, aged 40, with a partially cystic thyroid nodule (PCTN) for 5 years, presenting hypoechoic features, fine calcifications, and smooth borders in an ultrasound examination. According to the nomogram’s formula, this profile corresponds to a calculated score of approximately 165, indicating a malignancy probability of about 45%, illustrated in Figure 3. In such instances, clinicians may opt for a follow-up strategy, given the malignancy risk falls below 50%. However, our research has pinpointed a critical threshold at a total score of 161, representing a 40% malignancy probability in Figure 4 (7). Consequently, setting the model’s cutoff value at this point is crucial. For patients reaching this score, our study strongly recommends proactive intervention, significantly reducing the risk of misdiagnosis and ensuring optimal surgical interventions. Furthermore, two curves in the postoperative management of surgical procedures has demonstrated promising benefits. Notably, a study conducted by Zhao successfully established a nomogram predicting lateral lymph node metastasis of thyroid papillary carcinoma based on blood immune indexes, showcasing the potential of two curves to refine nomogram accuracy and facilitate informed clinical decisions (9).

## Discussion

Nomograms, valuable predictive tools widely used in cancer patient clinical studies, were a focal point of our research. Our study aimed to enhance the nomogram evaluation system, emphasizing precision medicine’s original intent: to deter misdiagnosis or overdiagnosis with an optimal cutoff value (10). Unfortunately, many studies overlook the critical selection process for this cutoff value, posing implementation challenges in clinical settings. Our research significantly contributes to refining and augmenting the evaluation of clinical models. We strived to identify an appropriate cutoff value, aligning with precision diagnosis, and aiming to mitigate misdiagnosis or over-diagnosis (11).

Due to the broad spectrum of clinical applications, our central goal is not only to pinpoint the optimal truncated value. In the realm of lung nodule screening, our model emphasizes heightened sensitivity, crucial for minimizing the risk of overlooking suspicious nodules (12). On the contrary, in the monitoring of infectious diseases, the model places greater emphasis on specificity, potentially raising concerns of misdiagnosis and harm to healthy patients (13). Thus, adapting the clinical model to align with the distinct needs of each clinical application becomes imperative. In our study, Figure 2 provides a visual representation of performance changes at different thresholds, aiding clinicians in making informed selections based on specific criteria depending on the context.

Although the curve proficiently illustrates the model’s performance variations at diverse thresholds, it is not without limitations. At the outset, it is not feasible to assess the superiority or inferiority of the model based on the alterations observed in the evaluation curve of the model’s predictive performance. Furthermore, there exists a dearth of a well-established fitting curve that pertains to the correlation between thresholds and total points of the nomogram, thereby necessitating the formulation of more optimal fitting equations. Moreover, caution is needed when any regression analysis was applied to actual clinical practice. Regression analysis provides tentative answers to complex questions, but these answers might not always have given an accurate representation. If statistical tools fall into the wrong hands, it could cause great harm to those involved. For example, because of been administered estrogen pills under the prescription of “for the patient’s health,” it increases the risk of stroke or premature mortality due to breast cancer among women (3).

In brief, nomograms can serve as a valuable tool for estimation and exploration in scenarios that demand expediency and involve a restricted number of variables. Our assertion is that these two curves can enhance the comprehension of the nomogram’s internal predictive capability and furnish additional direction for the clinical implementation of the predictive model. Nevertheless, their usage should be approached with circumspection and their limitations comprehended to prevent erroneous outcomes.

## Data Availability

The original contributions presented in the study are included in the article/supplementary material, further inquiries can be directed to the corresponding author.
